# Transcriptomics and Proteomics Analyses Reveal JAK Signaling and Inflammatory Phenotypes during Cellular Senescence in Blind Mole Rats: The Reflections of Superior Biology

**DOI:** 10.3390/biology11091253

**Published:** 2022-08-23

**Authors:** Nurcan Inci, Erdogan Oguzhan Akyildiz, Abdullah Alper Bulbul, Eda Tahir Turanli, Emel Akgun, Ahmet Tarik Baykal, Faruk Colak, Perinur Bozaykut

**Affiliations:** 1 Department of Molecular and Translational Biomedicine, Institute of Natural and Applied Sciences, Acibadem Mehmet Ali Aydinlar University, Istanbul 34752, Turkey; 2 Department of Biostatistics and Bioinformatics, Institute of Health Sciences, Acibadem Mehmet Ali Aydinlar University, Istanbul 34752, Turkey; 3 Department of Molecular Biology and Genetics, Faculty of Engineering and Natural Sciences, Acibadem Mehmet Ali Aydinlar University, Istanbul 34752, Turkey; 4 Department of Medical Biochemistry, Faculty of Medicine, Acibadem Mehmet Ali Aydinlar University, Istanbul 34752, Turkey; 5 Department of Biology, Faculty of Arts and Science, Zonguldak Bulent Ecevit University, Zonguldak 67100, Turkey

**Keywords:** aging, cancer, cellular senescence, JAK–STAT, SASP, inflammation, anticancer, MMP, NF-κB

## Abstract

**Simple Summary:**

Blind mole rats (BMR) (*Spalax, Nannospalax* sp.) are extraordinary organisms with cancer resistance and a long lifespan for their size. Cellular senescence is a condition in which cells cease dividing irreversibly and secrete proinflammatory cytokines. To understand the mechanisms behind their superior traits, we utilized transcriptomics and proteomics tools in senescent BMR cells to compare them to similarly sized mice. The results revealed the alterations in Janus kinase (JAK) signaling and the cytokine-mediated pathway during the cellular senescence process in BMRs. These findings might reveal the novel mechanisms behind the unique biology of BMRs through cytokine-mediated adaptations.

**Abstract:**

The blind mole rat (BMR), a long-living subterranean rodent, is an exceptional model for both aging and cancer research since they do not display age-related phenotypes or tumor formation. The Janus kinase–signal transducer and activator of transcription (JAK–STAT) signaling is a cytokine-stimulated pathway that has a crucial role in immune regulation, proliferation, and cytokine production. Therefore, the pathway has recently attracted interest in cellular senescence studies. Here, by using publicly available data, we report that JAK–STAT signaling was suppressed in the BMR in comparison to the mouse. Interestingly, our experimental results showed upregulated *Jak1*/*2* expressions in BMR fibroblasts during the replicative senescence process. The transcriptomic analysis using publicly available data also demonstrated that various cytokines related to JAK–STAT signaling were upregulated in the late passage cells, while some other cytokines such as MMPs and SERPINs were downregulated, representing a possible balance of senescence-associated secretory phenotypes (SASPs) in the BMR. Finally, our proteomics data also confirmed cytokine-mediated signaling activation in senescent BMR fibroblasts. Together, our findings suggest the critical role of JAK–STAT and cytokine-mediated signaling pathways during cellular senescence, pointing to the possible contribution of divergent inflammatory factors to the superior resistance of aging and cancer in BMRs.

## 1. Introduction

Aging is the progressive decline in the cellular and physiological function of the cells, leading to age-related pathologies such as neurodegenerative and cardiovascular diseases, diabetes, and cancer [1]. However, some organisms live long and are naturally resistant to age-related diseases. As such, blind mole rats (BMRs) (*Spalax* or *Nannospalax* spp.), which are subterranean rodents commonly found in the Middle East, are regarded as living a long time for their size, with a lifespan exceeding 21 years. Studies have shown that the BMR has also evolved a strong resistance against spontaneous and experimentally induced tumorigenesis [2,3]. Thus, BMRs represent a powerful model for studying mammalian adaptation to aging and cancer. 

Previous studies demonstrated that concerted cell death (CCD), a mechanism triggered by the secretion of interferon-β (IFN-β), is responsible for the anticancer resistance of BMRs [2]. It has been proposed that after 7–20 population doublings, the cells undergoing CCD begin to secrete IFN-β to prevent hyperplasia [2,4]. On the other hand, alterations in the immune system such as increased proinflammatory cytokines and chemokines are closely associated with the aging process [5,6]. This phenomenon is also called immunosenescence and represents one of the hallmarks of aging through a low-grade chronic inflammation status [7]. From this perspective, it is intriguing that BMRs display resistance to aging pathologies during the secretion of elevated cytokines such as IFN-β during increased population doublings.

Aging-related chronic inflammation has been suggested as being driven by cellular senescence, i.e., the stable arrest of cell growth [8]. Senescent cells accumulate with aging and harm the neighboring cells by the “the senescence-associated secretory phenotype (SASP)”, which includes the secretion of proinflammatory cytokines, chemokines, growth factors, and matrix metalloproteinases (MMPs) [9]. Then again, the Janus kinase–signal transducer and activator of transcription (JAK–STAT) is a signaling pathway that has a primary role in various biological processes including the production of cytokines such as IFN-β and interleukins [10,11,12].

JAK–STAT is a well-studied pathway in cancer [13], and recently, it has also gained interest in aging and cellular senescence studies [14]. However, its role in the adaptive mechanisms of the BMR remains unknown. Here, to fill this gap, we first analyzed publicly available RNA-Seq datasets and found that JAK–STAT signaling is suppressed in the BMR when compared to mouse transcriptomes. This led us to investigate the expression levels of JAK–STAT signaling in BMR fibroblasts during the cellular senescence process. To this end, we induced replicative senescence in BMR cells, which is represented by increased population doublings as a result of serial passaging. Consistent with the previous findings on the increased level of IFN-β at increased population doublings in BMR cells [2], we showed elevated *Jak1* and *Jak2* mRNA expressions. To complement our data, we reanalyzed the BMR and mouse RNA-Seq data of late passage cells. The results pointed to increased levels of major SASP components including *Il1α* and *Il1β*; however, it also suggested decreased levels of other SASP components such as MMPs. We also performed a comparative proteomic analysis in early and late passage BMR fibroblasts for protein identification and revealed an increased cytokine–cytokine-mediated signaling in senescent BMR cells. Overall, this study points out that JAK–STAT signaling could be connected to both the cancer and aging adaptations of the BMR through diverse cytokine expression profiles.

## 2. Materials and Methods

### 2.1. Animals

BMRs are extremely difficult to breed in laboratory conditions [15]. The ones used in this study were captured from nature in Bolu, Turkey, where a parallel, long-term study on their population structure and dynamics was being conducted for age determination by our group. The body weight, sex, health status, and approximate ages of the animals were recorded upon capture (male, 6–12 months old) (*n* = 4). Similarly aged C57BL/6 male mice were obtained from the animal facility of Acibadem University (male, 3–9 months old) (*n* = 4). The study was approved by the Ethics Committee of Acibadem University (#HDK-2020/12).

### 2.2. Fibroblast Isolation and Cell Culture

Fibroblasts were isolated from BMRs and mice as previously described [16]. Briefly, lungs were harvested and washed in sterile PBS to remove the remaining blood. Tissues were cut into small pieces in 2 mL of collagenase-A solution, transferred into a 15 mL tube, and digested for 4 h at 37 °C. Then, the collagenase-A solution was removed and Dulbecco’s modified Eagle’s medium (DMEM)/F12 with 15% fetal bovine serum (FBS) added, and the tissues were pipetted repeatedly to generate a cell suspension. The cells were then plated into cell culture plates and incubated in the standard incubator.

All BMR fibroblasts showed similar growth characteristics, and they were supplemented with DMEM/F12 media (ATCC) and with 15% FBS (Thermo Fisher, Waltham, MA, USA), nonessential amino acids, sodium pyruvate, and 1% penicillin–streptomycin (Thermo Fisher, Waltham, MA, USA). Primary mouse fibroblasts were cultured in DMEM (BioWest, Riverside, MO, USA) and supplemented with 10% FBS and 1% penicillin–streptomycin (Thermo Fisher, Waltham, MA, USA). Both mouse and BMR fibroblasts were incubated at 37 °C and 5% CO_2_, and cells were passaged after they reached confluence.

Both mouse and BMR fibroblasts were subjected to serial passaging to induce replicative senescence. Passages 2–3 (P2–3) represent early passages for mouse and BMR cells, P6–7 represent late passages for mouse cells, and P7–8 represent late passages for BMR cells for the rest of the study.

### 2.3. β-Galactosidase Staining

Cellular senescence was induced with replicative passaging in fibroblasts; early passage represents cells from Passages 2–3, and late passage represents cells from Passages 7–8. Then, 1.5 × 10^4^ cells from the early and late passages were simultaneously seeded to 96-well plates in triplicate. Cells were subsequently washed with phosphate-buffered saline (PBS) and fixed with paraformaldehyde for 10 min. After fixation, cells were washed with bovine serum albumin. All samples were dyed with the working solution from the CellEvent™ Senescence Green Detection Kit (Invitrogen Cat no: C10850, Thermo Fisher, Waltham, MA, USA). The plate was incubated at 37 °C for 2 h without CO_2_ to protect the pH of the solution. After that, cells were washed with PBS, and images of senescent cells were captured with a Zeiss Axio inverted fluorescence microscope (New York, NY, USA).

### 2.4. Cell Growth Curve

Cells from early and late passages were seeded at 4 × 10^4^ cells per well in a 24-well plate. Cells were harvested every 24, 48, 72, 96, and 120 h, and the number of cells was counted for the growth curve in triplicate.

### 2.5. RNA Extraction and qPCR

Total RNA from the early and late passages were extracted with a Zymo Quick-RNA MiniPrep kit (R1054, Zymo Research, Tustin, CA, USA) according to the manufacturer’s instructions. The RNA concentration and purity were evaluated with a NanoDrop One spectrometer (Thermo Fisher Scientific Inc., Waltham, MA, USA). The SensiFAST cDNA synthesis kit (BIO-65053, SensiFAST, Bioline, Memphis, TN, USA) was used to synthesize cDNA from RNA. Each PCR reaction was conducted in a 20 μL reaction mixture with a final amount of 10 ng cDNA for each mixture using the SensiFAST SYBR No-ROX qPCR kit (BIO-98005, Bioline, Memphis, TN, USA). Quantitative RT-qPCR experiments were carried out with the CFX96 Touch Real-Time PCR Detection System (Bio-Rad, Berkeley, CA, USA). The cycling parameters were as follows: 95 °C for 2 min, followed by 40 cycles at 95 °C for 5 s, an optimized annealing temperature for 10 s, and 72 °C for 5 s. The results of each target gene were normalized to β-actin mRNA expressions. The primer sets were designed using the Primer3 online software (https://primer3.ut.ee/) (accessed on 21 November 2021). The list of primers used for the experiments is shown in Table S1. Each experiment had three replicates per condition, and a statistical analysis was performed with a Mann–Whitney *t*-test using GraphPad Prism 9.3.1.

### 2.6. Protein Extraction and Nano LC-ESI-MS/MS Analysis

For protein extraction, samples were mixed with a universal protein extraction (UPX) buffer (Expedeon-44101) and, then, were sonicated with 5 × 10 sec cycles. After sonication, the samples were cooled on ice and incubated at 95 °C for 10 min. The samples were centrifuged at the maximum rpm for 10 min, and the supernatant was transferred to new tubes. The protein concentration was determined by the Bradford method. Peptide extraction was performed using the FASP Protein Digestion Kit (Expedeon-44250) and the trypsin enzyme (Pierce-90057). The peptide concentration was determined by the Pierce™ Quantitative Colorimetric Peptide Assay (Thermo-23275). The peptides were acidified and diluted to 200 ng/µL by 0.1% formic acid and transferred to liquid chromatography (LC) vials for injection.

The Acquity UPLC M-Class SYNAPT XeVo G2-XS system (Waters, Milford, MA, USA) was used for analysis. The mass spectrometry (MS) instrument was operated in positive ion mode. A novel data-independent mode, the SONAR method, was performed for data acquisition. The detector and calibration settings were set by the MassLynx program (V4.1; Waters, Milford, MA, USA), which is specific to the Xevo G2-XS Q-ToF (Waters, Milford, MA, USA) device on which we performed the analysis. Then, SONAR and the sensitivity mode were turned on, and the tryptic peptides were subjected to 120 min reverse phase chromatography at a flow rate of 300 nL/min in an HSS T3 (Waters-186,008,818) nano column. The peptides were separated from the column by an elevation in the range of 5–35% acetonitrile according to their hydrophobicity, and then, they were analyzed by mass spectrometry. Data were collected for peptides that could be identified in the m/z range of 50–2000. The MS analysis was performed for 0.5 s, and information was gained about the entire peptide. Then, the MS/MS analysis was performed for 0.5 s, and the peptide fragmentation and sequence information were collected. The quantitative analysis of peptide features and protein identification was performed with Progenesis QI for the proteomics software.

### 2.7. Data Analysis of LC MS/MS

A UniProt mouse and the *Nannospalax* databases were used for protein identification. The UniProt IDs of the obtained proteins were acquired from the text obtained with the pandas package of Python [17]. Using the R tximport package and the TxDb.Mmusculus.UCSC.mm10.knownGene database, the UniProt IDs were mapped to gene symbols and the Entrez ID. *Nannospalax* protein amounts obtained using the R edgeR package were normalized, and differential expression analyses were performed. Dot graphs of the set enrichment results were obtained with the clusterProfiler tool. Using Cytoscape EnrichmentMap and auto-annotation tools, network graphs with sets and annotations of the clustering results were created [18].

### 2.8. Reanalysis of RNA Sequencing and Proteomics Datasets

To identify the effect of replicative senescence across species including BMRs and mice, we used the following publicly available datasets from Gene Expression Omnibus (GEO): GSE181413 [4], GSE180008 [19], GSE117501 [20], GSE63577 [21], and GSE181733 [4]. Fastq data from the BMR sequencing (RNA-Seq) were taken from the GEO Database with NCBI in the SRA tool [22]. The quantification of the RNA-seq data was performed with the Salmon alignment free quantification tool [23]. BMR and mouse Fastq data were generated using the GENCODE release M28 (GRCm39) coding sequence. The R “org.Mm.eg.db” database and Tximport package were used for the annotation of transcripts [24,25,26]. Gene-based normalizations were performed with the R edgeR package [27]. Logarithmic fold changes of genes were calculated by the generalized linear model method and tested with a likelihood ratio test. The enrichment analyses were performed using the obtained gene fold changes with clusterProfiler and the GSEA tool, using KEGG, and the reactome and gene ontology gene sets [27,28]. A p-value of 0.05 was chosen as the gene set’s significance cutoff. Gene sets in each database were analyzed with 10,000 permutations. A multiple test correction was performed with the Benjamini–Hochberg procedure. The results were graphed with the ggplot2 and clusterProfiler tools [29]. To create heatmaps of the JAK–STAT signaling pathway, the R ComplexHeatmaps package was used with scaled raw gene expression abundance. 

### 2.9. Statistical Analysis

Data are represented as the mean ± SD. The statistical procedures and graph plotting were conducted in GraphPad Prism 9.3.1 (GraphPad Software, La Jolla, CA, USA). The means of the two groups were compared and analyzed using a non-parametric Mann–Whitney unpaired *t*-test. Differences were considered statistically significant when the *p*-value was less than 0.05.

## 3. Results

### 3.1. JAK–STAT Pathway Is Suppressed in the BMR Compared to the Mouse 

**Figure 1:**
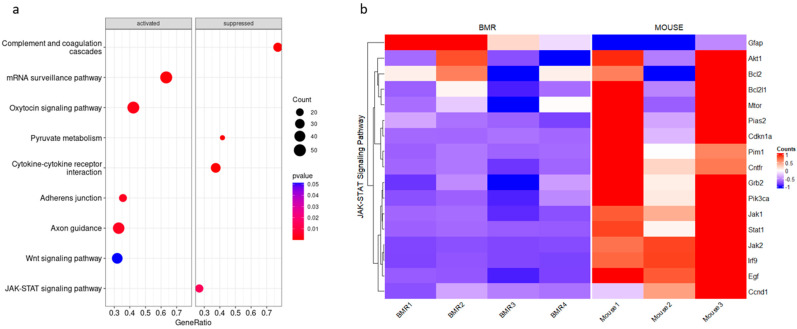
Comparison of differentially expressed genes between BMRs and mice (data were reanalyzed from the transcriptome data of Altwasser et al.). (**a**) KEGG pathway enrichment analysis of consistently activated and repressed genes in BMRs (*n* = 4) when compared to mice (*n* = 3). The permutation of the gene set enrichment analysis is 10,000, and the cutoff *p*-value was selected as 0.05. RNA samples from BMRs represent the sequence data. The dot size is based on the gene count enriched in the pathway, and the color of the dot shows the pathway enrichment significance. Gene set enrichment analyses were performed with multiple test corrections (Benjamini–Hochberg). (**b**) Expression changes in JAK–STAT signaling are represented as a heatmap by using the transcriptome data of BMRs and mice. The columns represent individuals, and rows represent genes. Before creating the heatmap, raw gene expression abundances were scaled at the gene level, which is represented as the Z-score.

BMRs display extraordinary traits as they have a much longer lifespan and remarkable cancer resistance when compared to similarly sized mice. Although revealing the underlying mechanisms of the BMR in comparison to the mouse is of great interest, these mechanisms have not been fully elucidated yet. Therefore, we first analyzed the available RNA-Seq data that belong to the adult BMR and mice samples [15]. After excluding the unannotated and duplicated genes, a differential expression gene (DEG) analysis was performed. A total of 7548 genes were differentially expressed in the BMR when compared to the mouse. Functional enrichment in the KEGG pathways and alterations in other signaling pathways including JAK–STAT signaling and cytokine–cytokine receptor suppression in BMR cells were compared with mouse cells, and the results are presented in Figure 1a (a full list of significantly regulated KEGG pathways is presented in Table S2). A further analysis indicated a total of 22 genes specific to the JAK–STAT signaling pathway (Figure 1b, Figure S1a). The most enriched genes that were downregulated in BMR cells included *Jak1*, *Jak2*, *Stat1*, *Ctf1*, *Cdkn1a (p21)*, *Irf9*, *Pik3ca*, and *Jak2*; *Jak2*, in particular, was one of the highest-level downregulated genes. However, these findings are based on health status and do not represent the aging process. Therefore, to observe the alterations in the JAK–STAT pathway during cellular senescence, we investigated their expressions in early and late passage fibroblasts.

### 3.2. Jak1 and Jak2 Levels Are Elevated in the BMR during Replicative Senescence

Since JAK–STAT signaling is found to be suppressed in BMR tissues according to the transcriptome data, we investigated the *Jak1* and *Jak2* mRNA expressions during the cellular senescence process in fibroblasts. For this, fibroblasts were subjected to serial passages to achieve increased population doubling and the senescent phenotype. Consistent with the previous study [16], *Nannospalax* fibroblasts entered replicative senescence during Passages 7–8, as shown in Figure S2. The senescence status of BMR cells was validated by the enlarged, flattened morphology (Figure S2a), the exhibition of positive SA-β-Gal staining (Figure S2b), and the halting of the proliferation demonstrated by the growth curve (Figure S2c). mRNA expressions of *Il6 and p16* in BMR cells with early and late passages were also analyzed (Figure S2d) (not significant). For the *p16* analysis, we used the *p15ink4b* sequence, since the *p16* sequence could not be found for *Nannospalax* (Table S1).

**Figure 2:**
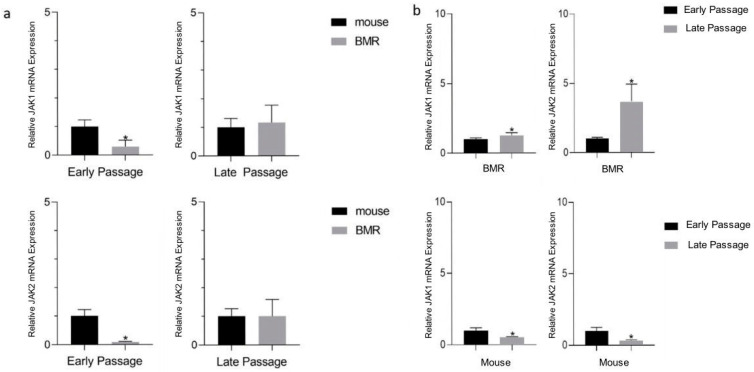
*Jak1* and *Jak2* mRNA expressions are increased in BMRs during replicative senescence. (**a**) *Jak1* and *Jak* gene expressions were compared between mouse and BMR cells either in early or late passages. (**b**) *Jak1* and *Jak2* gene expressions were compared in early and late passage cells in either mice or BMRs. The expression levels were quantified with qRT-PCR. The *p*-value was calculated with a Mann–Whitney test (*n* = 3, * *p* < 0.05), and the standard deviation (SD) represents the mean expression values of three replicates.

In addition, to complement the RNA-Seq data (Figure 1) [15], we analyzed *Jak* mRNA expressions in early and late passage fibroblasts belonging to both BMRs and mice, as shown in Figure 2. We first comparatively analyzed *Jak1* and *Jak2* mRNA expressions between mice and BMRs either in early passage or late passage fibroblasts (Figure 2a). Our results showed that although *Jak1* and *Jak2* expressions were lower in the early passages of the BMRs when compared to the mice, surprisingly, no significant change was detected between the late passages (Figure 2a). To better understand this result, we then compared early and late passage cells separately either in mice or in BMRs. We found that mRNA expressions were significantly increased during the replicative senescence process in BMR fibroblasts as opposed to mouse fibroblasts (Figure 2b), suggesting a rational explanation to the unsignificant change in the late passage cells between mice and BMRs (Figure 2a). In addition, the upregulation of JAK signaling in BMRs during replicative senescence also supports the previous study on the activation of the IFN pathway in the late passage BMR cells undergoing CCD [4], since the JAK pathway is known to induce the IFNs. On the other hand, no significant change was found in the *Stat3* gene expression in BMRs with replicative senescence (Figure S3).

### 3.3. Divergent Action of Inflammatory Factors during Cellular Senescence

To further study the underlying mechanisms related to replicative senescence in BMRs, we also reanalyzed the publicly available RNA-Seq data from the early and late passage BMR fibroblasts [4]. Following the removal of unannotated and duplicated genes, the DEG analysis showed that a total of 3633 genes were differentially expressed in the BMR late passage cells compared to the BMR early passage cells. These genes were enriched in the KEGG pathway; activated pathways in late passages included proteasome, nucleotide excision repair, the glutathione metabolism, the cell cycle, and cellular senescence, all of which are closely related to the aging process (Figure S4a). Regarding the direct relationship of cellular senescence with this study, a detailed KEGG graph for this pathway is given in the Supplementary Data (Figure S4b). The KEGG analysis demonstrated that other immune-system-related pathways including the cytokine–cytokine receptor interaction and the nuclear factor-kappa B (NF-κB) signaling pathway were also elevated in the late passage BMR cells (Figure S4a). On the other hand, the main pathways such as mTOR and insulin signaling were suppressed in late passage cells (Figure S4a). A full list of significantly regulated KEGG pathways can be found in Table S3. 

**Figure 3:**
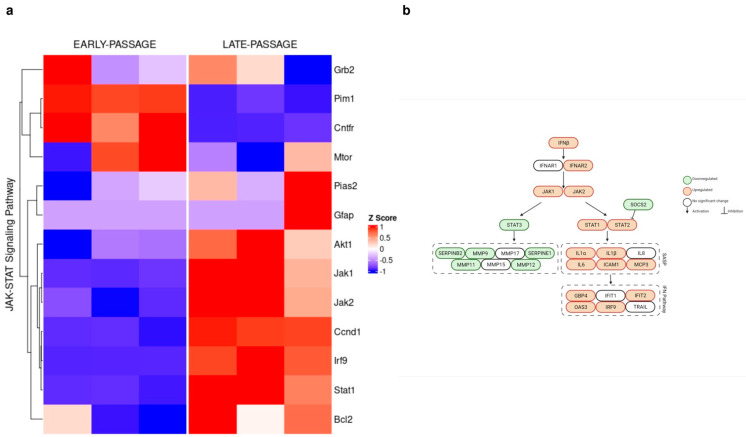
Differentially expressed genes between early and late BMR cells are compared. (**a**) A KEGG heatmap for the genes related to JAK–STAT signaling is shown. Upregulated and downregulated gene expressions in late BMR cells are compared to early BMR cells. The permutation of the gene set enrichment analysis is 10,000, and the cutoff *p*-value was selected as 0.05. The gene set enrichment scores were performed with a multiple test correction (Benjamini–Hochberg) (*n* = 3). Before creating the heatmap, raw gene expression abundances were scaled at the gene level, which is represented as the Z-score. (**b**) A schematic overview of the JAK–STAT pathway and SASP is represented with downregulated and upregulated genes.

In addition, significantly changed genes related to JAK–STAT signaling in BMRs are plotted as a heatmap (Figure 3a). Specifically, the upregulation of *Akt*, *Irf9*, *Stat1*, *Jak1*, *Jak2*, and *Ccnd1* was observed in late passage BMR cells, which is consistent with our RT-qPCR results. A schematic overview of the changes in the expression levels and the relationship between JAK–STAT pathway genes and IFN signaling is represented in Figure 3b. IL-6 expression was increased with senescence in the RNA-Seq data; however, we could not detect a significant change in the qPCR results. This might be due to the low expression of IL-6, which was also discussed in the previous study [16].

Moreover, we also analyzed the RNA-Seq data in senescent BMR and mouse cells compared to their younger cells in terms of SASP and JAK–STAT signaling. The significant changes according to the comparative RNA-Seq analysis are shown in Table 1. Cytokines such as *Ifnβ*, *Il1β*, *Il1α*, and *Icam1* were elevated; however, some other inflammatory factors related to SASP in the cellular senescence process such as *Mmp9*, *Mmp11*, *Mmp12*, *Serpine1*, and *Serpinb2* were downregulated in the senescence status of BMRs (Table 1).

Finally, we performed an analysis of the publicly available transcriptomic data belonging to mouse and human early and late passage fibroblasts in addition to those from BMRs (Figure S5). Although we detected immune-system-related changes in the late passages, those were different from the BMR alterations, suggesting that the BMR may follow different mechanisms from mice and humans during the aging process.

### 3.4. Proteomics Analysis Showed the Upregulation of the Cytokine-Mediated Signaling Pathway in BMRs during Replicative Senescence

**Figure 4:**
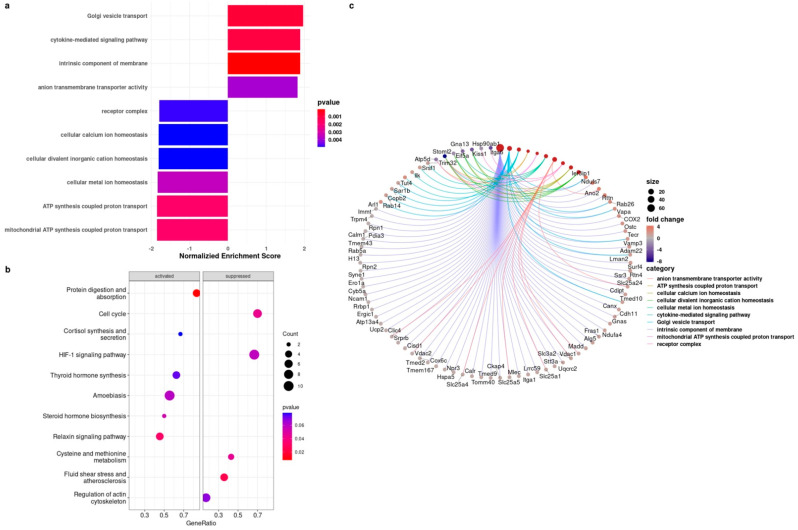
Proteomics analysis of the early and late passages of BMR cells. (**a**) The bar graph illustrates the pathway enrichment visualization process overrepresented in the related pathways in late passage cells compared to early passage cells with a normalized enrichment score. (**b**) A dot plot representation of the KEGG pathway enrichment analysis shows the activated and suppressed biological processes in late passage cells compared to early passage cells. The dot size is based on the gene count enriched in the pathway, and the color of the dot shows the pathway enrichment significance. Gene set enrichment analyses (GSEAs) were performed with multiple test corrections (Benjamini–Hochberg). (**c**) The Cnet plot of the BMR proteomics analysis is shown. Each line shows the pathway and gene association. The fold changes of proteins are colored between red and blue. The gene ratios of the pathways are presented as the size of a circle. Pathways were selected as statistically significant at the *p* < 0.05 cutoff (*n* = 4).

To extend our analysis beyond mRNA expression, we also investigated the alterations in the protein expressions in late passage BMR cells compared to early passage BMR cells by LC MS/MS. The proteomics results were correlated with our findings, and the pathways enriched in Gene Ontology (GO) included the upregulation of cytokine-mediated signaling pathways in senescent BMR fibroblasts (Figure 4a). Proteins identified in the enriched cytokine-mediated signaling pathway included terminal uridylyltransferase 4 (TUT4), integrin-linked protein kinase (ILK), serine-/arginine-rich splicing factor 1 (SRSF1), and E3 ubiquitin-protein ligase (TRIM32). Moreover, the main biological pathways that were either activated or suppressed in late passage cells by the KEGG pathway enrichment analysis are shown in Figure 4b. Among them, the highest enriched pathways were HIF1 signaling and cell cycle pathways. A full list of significantly regulated KEGG and GO pathways of proteomics data can be found in Table S4 and the full list of the identified proteins in early and late BMR fibroblasts is also given in Table S5. A detailed and specific pathway analysis was also performed in order to compare the changes in the related proteins belonging to the significantly regulated pathways (Figure 4c).

## 4. Discussion

The BMR is a model organism with unique properties related to longevity and cancer; therefore, the species presents great importance for attaining a better understanding of human aging [30]. The aging process is closely related to defects in the immune system such as alterations in the immune cell subset function and decreased B and T cell proliferation, resulting in a chronic low-grade inflammation status [31,32]. That inflammation status is related to the SASP, which progressively changes tissue homeostasis and contributes to the aging-related phenotype [33]. Here, in the present study, our reanalysis of the existing transcriptome data showed that *Jak1* and *Jak2* expressions were lower in adult and healthy BMRs when compared to mice and, thus, pointed to the potential effect of JAK signaling in the extraordinary aging traits of BMRs.

However, when BMR fibroblasts were subjected to serial passaging and entered replicative senescence, *Jak1* and *Jak2* mRNA expressions were surprisingly shown to be increased. In addition, we also comparatively analyzed *Jak1* and *Jak2* across BMR and mouse fibroblasts. Although *Jak* mRNA levels were lower in the BMR compared to mice with a non-senescent status, there was no significant difference following the cellular senescence process, due to the increased *Jak* levels in the senescent BMR cells.

To better understand the action of the JAK–STAT pathway and cytokine regulation in BMRs, we also compared whole-transcriptome RNA-Seq data belonging to early and late passage cells of BMRs [4,34]. Transcriptomics data showed that genes related to JAK–STAT signaling were activated in senescent BMR cells along with the cytokine–cytokine receptor interaction and NF-κB pathways. In addition, the RNA-Seq data also provided evidence for significantly increased cytokines such as *Ifnβ*, *Il1β*, *Il1α*, and *Icam1*. On the other hand, various *Mmp* and *Serpin* expressions were shown to be decreased in the senescent BMR cells. These RNA-Seq results are plausible, as the BMR also has a striking cancer resistance. Previous studies proposed that the BMR resists tumor formation through the CCD mechanism, which is triggered by IFNβ secretion, and that IFNβ mRNA expression was significantly increased before entering CCD in the late passage senescent cells [4]. Therefore, the discovery of the divergent expressional status among the cytokines may suggest that some cytokines could play a role in the anti-tumor mechanism, while others may play a role in the aging process. Other than the cytokines, MMPs and SERPINs are also considered to be important players in the SASP, and elevated protease secretion contributes to chronic inflammation [35,36].

In particular, the RNA-Seq data showed reduced *Mmp9*, *Mmp11*, *Mmp12*, *Serpine1*, *Serpinb2*, as well as *IL10* mRNA expressions in senescent BMR cells. MMPs are the family of proteases with a role in the regulation of the extracellular matrix (ECM), and ECM alterations are known to be critical during the aging process [37]. The studies in human and mouse fibroblasts under replicative or stress-induced senescence showed the increased secretion of a large number of MMPs [38], suggesting that the MMP family is an important SASP component in human and mouse senescence biology. In addition, SERPINE1 (also known as plasminogen activator inhibitor-1 (PAI-1)) is a well-known cellular senescence mediator and has also been shown to be a prominent biomarker in a recent proteomic SASP ATLAS study [39]. Our data provided evidence that, during the cellular senescence process, in contrast to human and mouse cells, BMR cells have lower MMP and SERPINE expressions and, thus, may receive protection from replicative senescence by balancing the SASP through divergent components of the SASP.

Other than the RNA-Seq data, proteomics analysis has not been performed in BMR cells representing cellular senescence status. Therefore, we performed an LC MS/MS analysis for a broader understanding of BMR senescence biology, for the first time. The identification of differentially expressed proteins in senescent BMR fibroblasts also pointed to the importance of the cytokine-mediated signaling pathway as an enriched pathway in GO analysis. More in particular, enriched proteins were related to IL6-mediated signaling (TUT4), IL17-mediated signaling (SRSF1, TRIM32) [40,41], and NF-κB signaling (ILK, TRIM32) [42,43,44]. TUT4 is an uridylyltransferase protein that is responsible for common cellular uridylation. It has been previously shown that TUT4 inhibits the silencing of IL6 and promotes cytokine expression [45]. Another significant protein that was found to be elevated during replicative senescence is ILK, which is an integrin-binding protein related to several cellular processes such as ECM degradation and, thus, also targets cellular senescence [46].

The activation of NF-κB signaling in senescent BMR cells was also partly shown in the proteomics data, consistent with the transcriptomic analysis. These data suggest the crucial role of the NF-κB pathway, which is also a major inducer of the SASP. Moreover, inflammatory cytokines stimulate NF-κB signaling in senescent cells and, therefore, may lead to the secretion of inflammatory factors contributing to the SASP, mainly through the IL-1β-stimulated autocrine IL-6 loop. In addition, NF-κB-related inflammation can also be induced by p38 MAPK, affecting IL-6, IL-8, and MCP1 activation [47]. Overall, proteomics data suggested that the activation of various proteins that play a prominent role in cytokine signaling can contribute to the SASP process in the replicative senescence of the BMR.

## 5. Conclusions

Our study revealed that JAK signaling is lower in BMRs compared to mice when their status is healthy. However, it is activated as a part of the cellular senescence process, possibly leading to cytokine elevation as an adaptation to the anti-tumor mechanism of CCD. On the other hand, some other SASP components are reduced during replicative senescence, pointing to the fact that BMRs may balance inflammatory status while resisting the age-related phenotype. 

Therefore, this study might provide evidence for the fact that since the BMR is a cancer-resistant animal in addition to having a long lifespan, it may have evolved different mechanisms for cancer and aging in terms of cytokine-related signaling. This affects the SASP through various inflammatory factors. Since aging is one of the main risk factors for the development of cancer, the present study gives a different point of view, allowing us to benefit from the superior biology of BMRs. However, in this study, protein fragments were identified by LC-MS/MS analysis mainly through the Mus musculus protein database, and only a portion of the genome of *Nannospalax* has so far been sequenced. Therefore, most of the genes and proteins were predicted computationally, which is why the results need to be validated in further studies.

## Figures and Tables

**Table 1:** Gene list related to cellular senescence/SASP and JAK–STAT signaling in the early and late passages of BMRs and mice.

Genes	BMR	Mouse	*p*-Value of BMRs	*p*-Value of Mice
*Mmp12*	↓	−	2.09 × 10^−3^	7.93 × 10^−1^
*Serpine1*	↓	↑	9.18 × 10^−29^	1.29 × 10^−3^
*Mmp11*	↓	↑	8.69 × 10^−1^	2.24 × 10^−2^
*Mmp9*	↓	↓	3.32 × 10^−124^	2.09 × 10^−2^
*Serpinb2*	↓	↓	3.96 × 10^−28^	4.68 × 10^−26^
*Il1β*	↑	−	1.95 × 10^−23^	7.64 × 10^−1^
*Il1α*	↑	↓	1.33 × 10^−236^	6.78 × 10^−8^
*Icam1*	↑	−	1.42 × 10^−11^	4.88 × 10^−1^
*Ifnβ*	↑	−	8.11 × 10^−3^	−
*Il10*	↓	−	8.64 × 10^−11^	2.78 × 10^−1^
*Jak1*	↑	−	1.31 × 10^−2^	2.83 × 10^−1^
*Jak2*	↑	−	3.50 × 10^−1^	8.41 × 10^−1^
*Stat1*	↑	↑	2.23 × 10^−1^	6.84 × 10^−3^
*Stat2*	↑	−	2.02 × 10^−12^	2.28 × 10^−1^
*Stat3*	↓	−	5.96 × 10^−7^	3.44 × 10^−1^
*Stat4*	↓	↑	8.64 × 10^−11^	4.94 × 10^−3^
*Stat6*	↓	↓	2.40 × 10^−5^	3.40 × 10^−2^

Abbreviations: ↑ significantly increased, ↓ significantly decreased, − not significant (the cutoff *p*-value for significancy was selected as 0.05) in the late passage cells of BMRs (*n* = 3 for early and late passages) or mice (*n* = 3 for early passage and *n* = 4 for late passage).

## Data Availability

The accession numbers of the RNA-Seq and proteomics data obtained from the Gene Expression Omnibus (GEO) are stated throughout the manuscript. The proteomics data are submitted as Table S5.

## Supplementary Materials

The following Supporting Information can be downloaded at: https://www.mdpi.com/article/10.3390/biology11091253/s1. Figure S1: Upregulated and downregulated JAK/STAT pathway-related genes in healthy BMRs compared to healthy mice are represented. Figure S2: BMR cells undergoing replicative senescence. Figure S3: The qRT-PCR results between the early and late passage of BMR and mouse fibroblasts are shown. Figure S4: The KEGG enrichment pathway for cellular senescence is represented in late passage BMR cells compared to early passage cells retrieved from RNA-Seq data. Figure S5: The dot plot KEGG enrichment map and GSEA plot show the significantly overrepresented pathways from the RNA-Seq data of mice and humans. Table S1: Primers designed for the RT-qPCR analysis. Table S2: A full list of the comparatively regulated KEGG pathway in healthy mouse and BMR samples. Table S3: A full list of the comparatively regulated KEGG pathway in the early and late passage of BMR samples. Table S4: comparatively regulated KEGG and GO enrichment analysis in the early and late passage of BMR proteomics data Table S5: A full list of the identified proteins in the proteomics analysis in the early and late passage BMR fibroblasts.

## Abbreviations

BMR	Blind mole rat
CCD	Concerted cell death
CDKN1a	Cyclin-dependent kinase inhibitor 1A
DEG	Differential expression gene
DMEM	Dulbecco’s modified Eagle’s medium
ECM	Extracellular matrix
GEO	Gene Expression Omnibus
GO	Gene Ontology
GSEA	Gene set enrichment analysis
HIF	Hypoxia-inducible factor
ICAM1	Intercellular adhesion molecule 1
IFNβ	Interferon β
IL17	Interleukin 17
Il1α	Interleukin 1 alpha
Il1β	Interleukin 1 beta
IL6	Interleukin 6
ILK	Integrin-linked protein kinase
IRF9	Interferon regulatory factor 9
JAK	Janus kinase
KEGG	Kyoto Encyclopedia of Genes and Genomes
LC-ESI-MS/MS	Liquid chromatography/electrospray ionization tandem mass spectrometry
MCP	Monocyte chemotactic protein
MMP	Matrix metallo-proteinases
mRNA	Messenger ribonucleic acid
NF-κB	Nuclear factor kappa B
p38 MAPK	p38 mitogen-activated protein kinases
PBS	Phosphate-buffered saline
PIK3CA	Phosphatidylinositol-4,5-bisphosphate 3-kinase catalytic subunit alpha
qRT-PCR	Quantitative real-time PCR
SASP	Senescence-associated secretory phenotype
SA-β-Gal	Senescence-associated β-galactosidase
SD	Standard deviation
SERPINs	Serine protease inhibitors
SRSF1	Serine-/arginine-rich splicing factor 1
STAT	Signal transducer and activator of transcription
TRIM32	E3 ubiquitin-protein ligase
TUT4	Terminal uridylyltransferase 4
