# Supplementary material for: Transcriptomics and Proteomics Analyses Reveal JAK Signaling and Inflammatory Phenotypes during Cellular Senescence in Blind Mole Rats: The Reflections of Superior Biology
Source: Biology (Basel). 2022 Aug 23;11(9):1253. doi: 10.3390/biology11091253 (PMC9495822; doi:10.3390/biology11091253)
Supplement: Supplementary file 1 [file biology-11-01253-s001.zip › Supplementary figures_revised.pdf]

Supplementary Figures

a

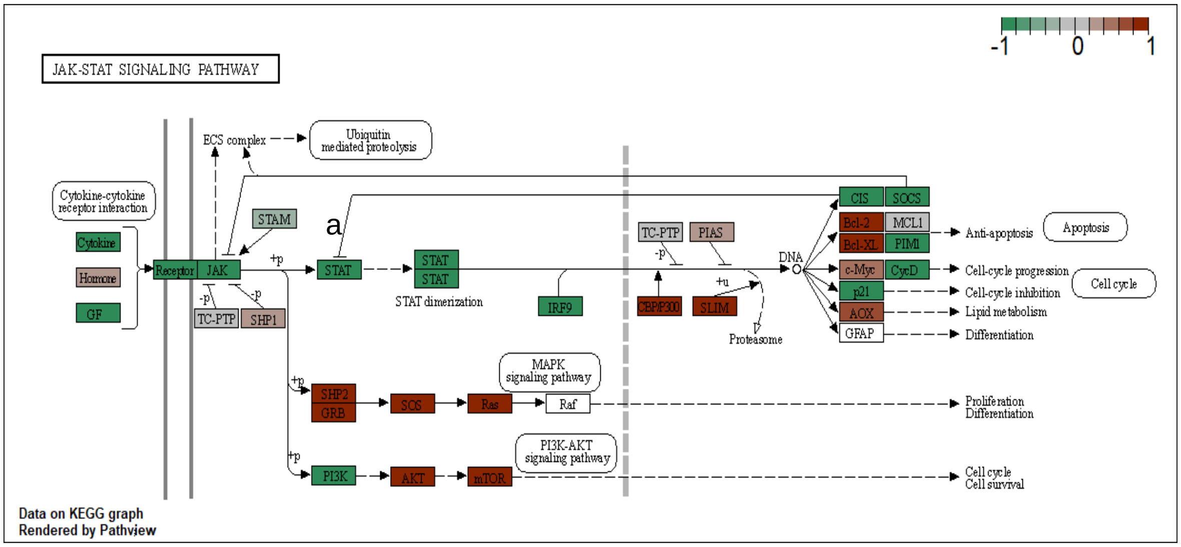

b

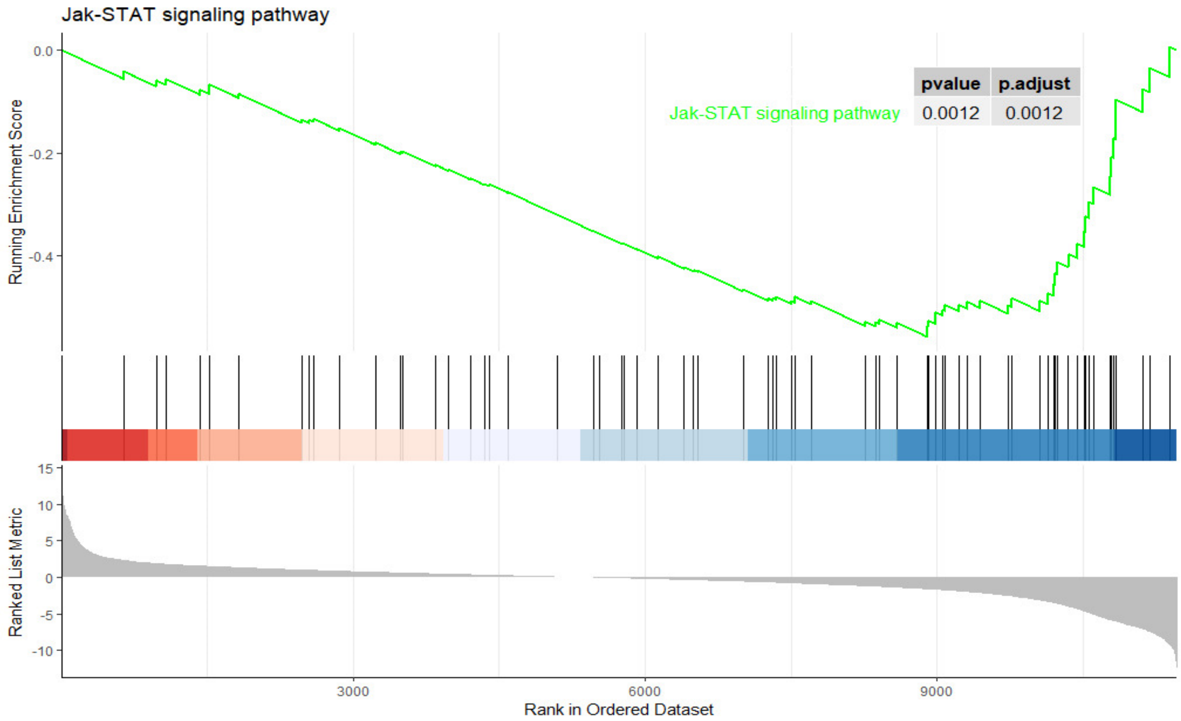

Supplementary Figure S1. a) Upregulated and downregulated JAK-STAT pathway related genes in healthy BMRs compared to healthy mouse are represented. b) Gene Set Enrichment Analysis (GSEA) enrichment score curves for JAK-STAT signaling pathway of BMR samples compared to mouse samples.

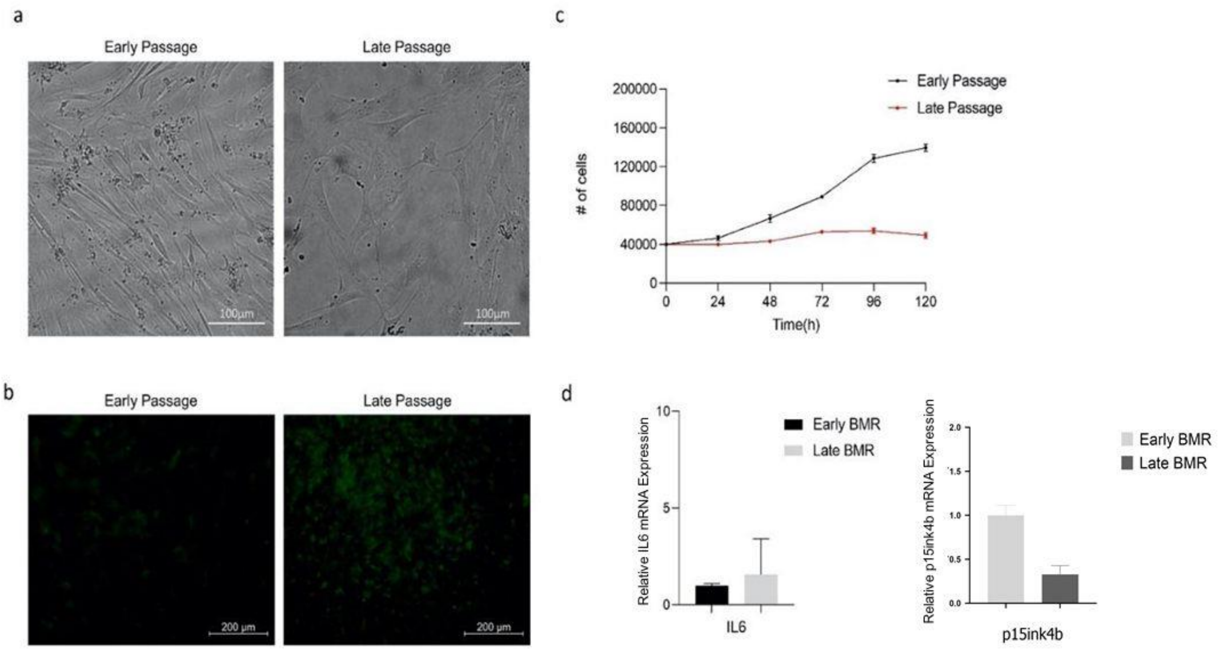

Supplementary Figure S2. BMR cells undergoing replicative senescence. (a) Morphological changes are observed between early and late passage cells. BMR fibroblast cells were seeded at 5 x 105 cells per T25 flask, and each image was photographed on day 3 (Lens, 20X. Bar, 100 µm). (b) Fluorescence images of early and late passages cells showing SA-β-gal staining. While SA-β-gal staining was observed in late passage cells, staining was not detectable in early passage cells (Lens, 10X. Bar, 200 µm). (c) Cell growth curve was compared between early and late passage cells. The experimentally observed cellular growth curve is shown by the number of the cells that were passaged every 24, 48, 72, 96, and 120 hrs. Data represents the number of counted BMR fibroblast cells. (d) mRNA Expressions of senescence biomarker IL6 and p15ink4b were compared in early and late BMR fibroblast cells. The expression levels were quantified with qRT-PCR and representative data for IL6 is shown. Three independent experiments repeated and bars represent mean ± standard deviation (SD). The fold change and SD are calculated from the mean expression values of three replicates, (n=3, *$p < 0.05$).

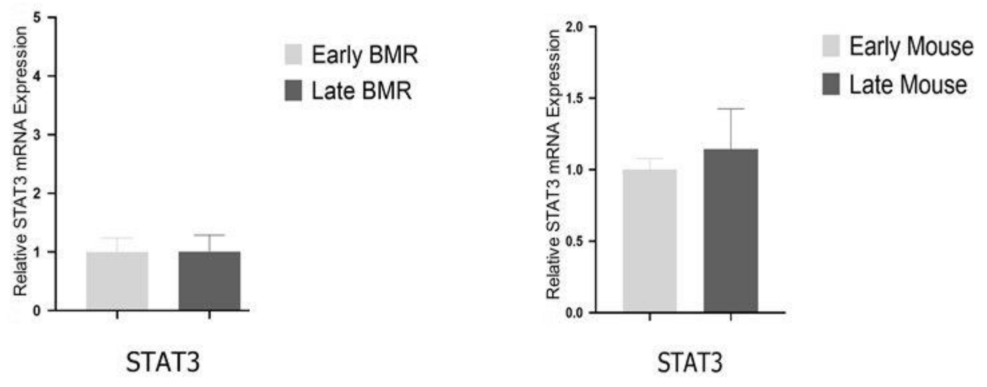

Supplementary Figure S3. qRT-PCR results between early and late passage of BMR and mouse fibroblasts are shown. Expressions of STAT3 are represented between early and late passage of BMR and mouse fibroblast cells. The expression levels were quantified with qRT-PCR. *p* value is calculated with Mann-Whitney test (n=3, *BMR;* Blind mole-rat). Calculations for the fold change and standard deviation (SD) are done with the mean expression values of three replicates. Three independent experiments repeated and bars represent mean ± *SD*, (n=3, *$p < 0.05$).

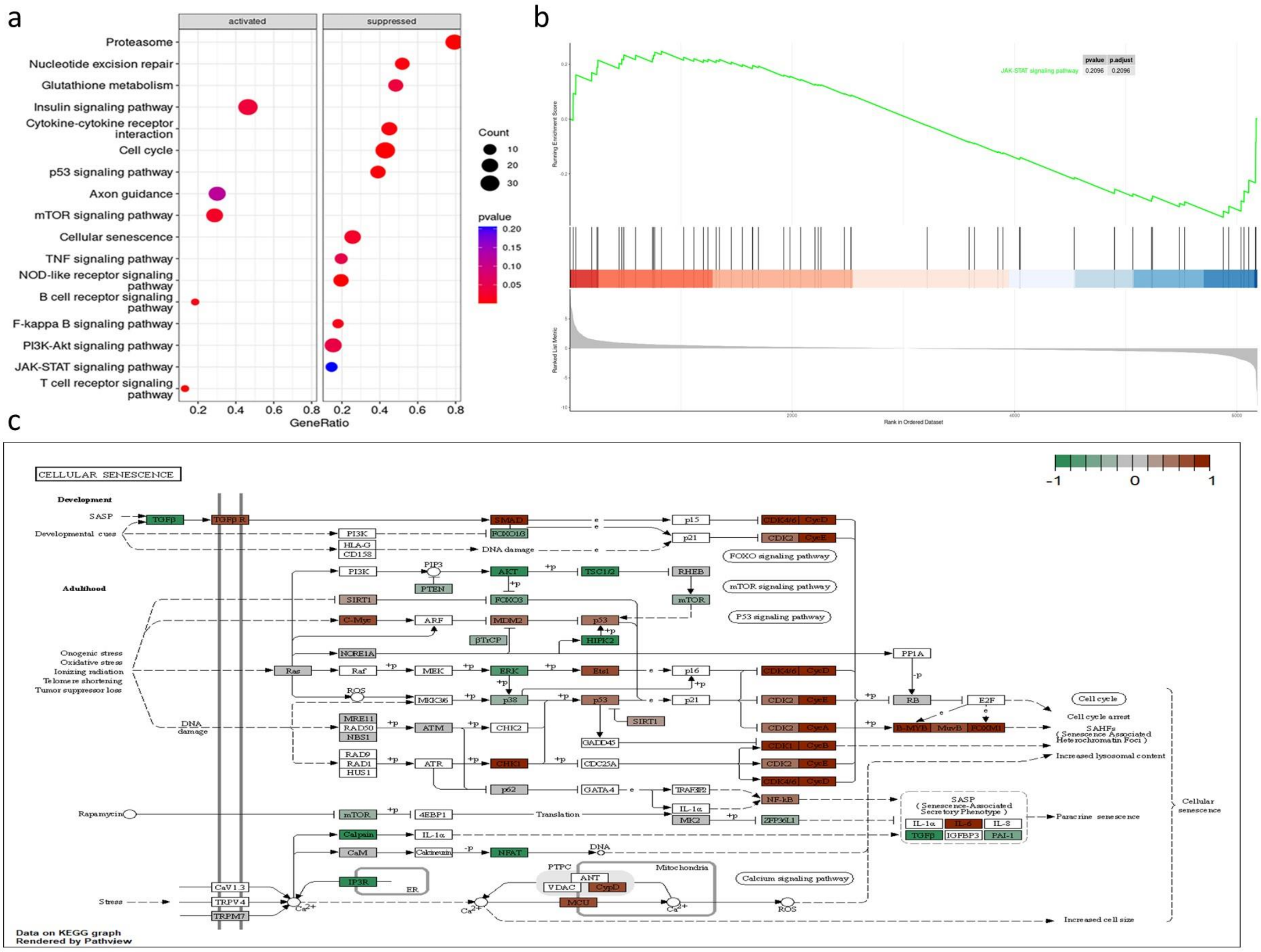

Supplementary Figure S4. (a) Dot plot of KEGG pathway enrichment analysis shows the activated and suppressed biological processes in late passage BMR compared to early passage BMR cells. The dot size is based on gene count enriched in the pathway, and the color of the dot shows the pathway enrichment significance. Permutation of gene set enrichment analysis is 10,000 and cut-off *p* value selected as 0.05. Gene set enrichment scores are performed with multiple test corrections (Benjamini-Hochberg) (n=3). b) KEGG enrichment pathway for cellular senescence is represented in late passage BMR cells compared to early passage cells retrieved from RNA-Seq data. c) Gene Set Enrichment Analysis (GSEA) enrichment score curves of JAK STAT signaling pathway of late passage BMR cells.

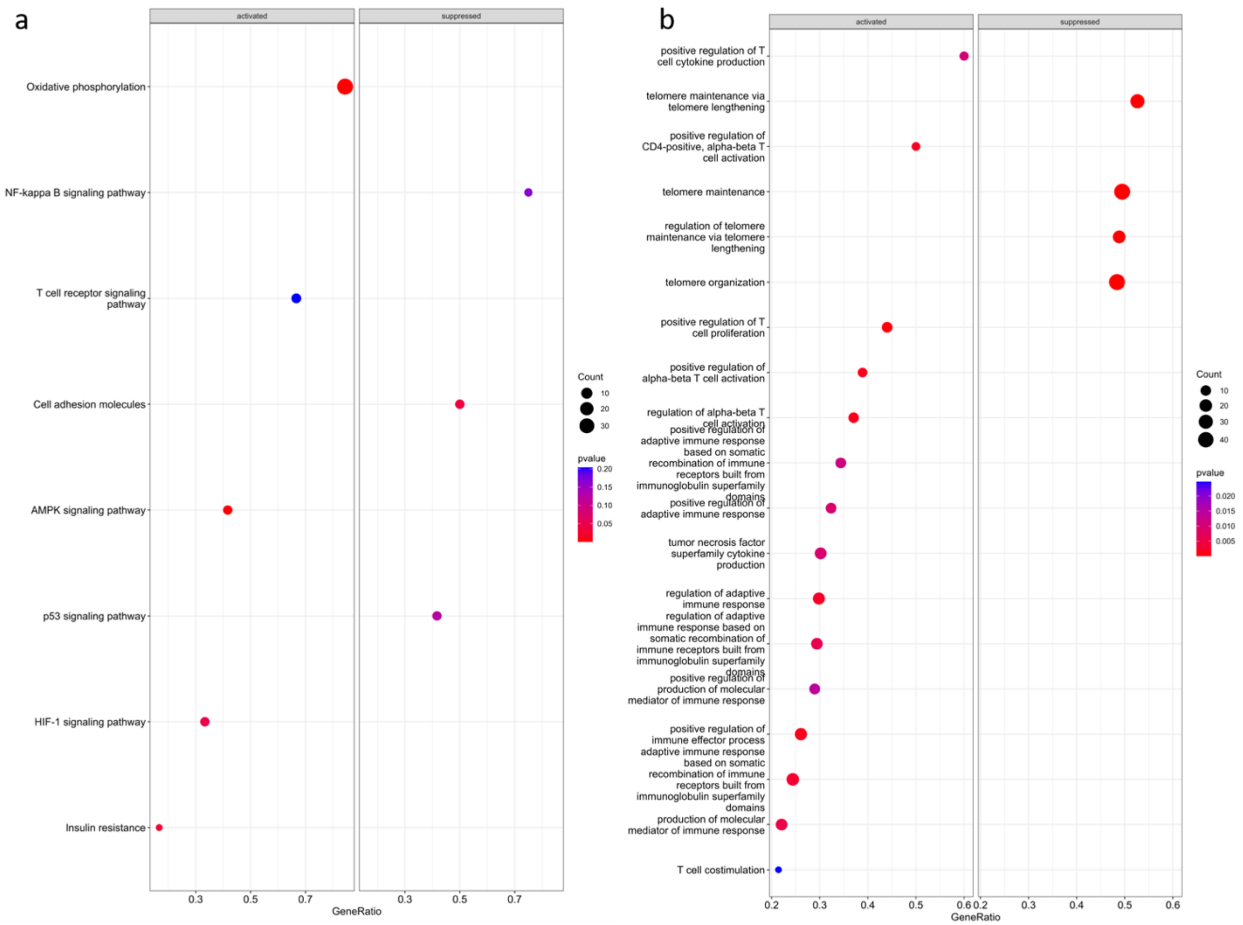

Supplementary Figure S5. Dotplot KEGG enrichment map shows the significantly overrepresented pathways from RNA-Seq data of mouse and human. Permutation of gene set enrichment analysis is 10,000 and cut-off *p* value selected as 0.05. Gene set enrichment scores are performed with multiple test corrections (Benjamini-Hochberg). (a) Representative data shows activated and suppressed pathways in late passage mouse cells compared to early mouse cells (n=4 for early passage, n=3 for late passage cells). (b) Representative data shows activated and suppressed pathways of late passage human fibroblast cells compared to early passage cells (n=18 for early passage, n=26 for late passage cells). The dot size is based on gene count enriched in the pathway, and the color of the dot shows the pathway enrichment significance.
