# Supplementary material for: Transcriptomics and Proteomics Analyses Reveal JAK Signaling and Inflammatory Phenotypes during Cellular Senescence in Blind Mole Rats: The Reflections of Superior Biology
Source: Biology (Basel). 2022 Aug 23;11(9):1253. doi: 10.3390/biology11091253 (PMC9495822; doi:10.3390/biology11091253)
Supplement: Supplementary file 1 [file biology-11-01253-s001.zip › Table S1.pdf]

Supplementary Table S1: Primers designed for RT-qPCR analysis.

Species	Gene	Left Primer	Right Primer
Blind mole-rat	JAK1	AATCTTCTTCTGGCCCGTGA	AGCAGCCACACTCAGATTCT
	JAK2	GGCAATGACAAGCAAGGACA	TGAAGGAGGGACGCTGATTT
	STAT3	CTTAAGCAGCCAAACCCCAG	CCCATCACAGGCTCAGAGAA
	IL6	GCTTGAACAATGACGAGGCC	GACATTGGCCTGCACGTTTT
	p15ink4b	GTGAAGTTCCAGGTGCCCTT	GTGTGTGTGTGGGGAAATGC
	B-actin	CCACCATGTACCCAGGCATT	CGGACTCATCGTACTCCTGC
Mouse	JAK1	TCTGTCACAACCTCTTCGCC	CATCAAGGAGTGGGGTTGCT
	JAK2	AGACGAGTCAACCAGGCATG	TAACACCGCCATCCCAAGAC
	STAT3	GGGCCTGGTGTGAACTACTC	TGAAGCGCAGTAGGAAGGTG
	B-Actin	CCACCATGTACCCAGGCATT	CGGACTCATCGTACTCCTGC
